# Novel Biotinylated Cu(II)-Phenanthroline Complexes: 2D and 3D Cytotoxic Activity and Mechanistic Insight

**DOI:** 10.3390/molecules28104112

**Published:** 2023-05-16

**Authors:** Stephen Barrett, Michele De Franco, Chiara Donati, Cristina Marzano, Valentina Gandin, Diego Montagner

**Affiliations:** 1 Department of Chemistry, Maynooth University, W23 NPY6 Maynooth, Ireland; stephen.barrett@mu.ie; 2 Department of Pharmaceutical and Pharmacological Sciences, University of Padova, 35131 Padova, Italy; michele.defranco@phd.unipd.it (M.D.F.); chiara.donati.1@phd.unipd.it (C.D.); cristina.marzano@unipd.it (C.M.); 3 Kathleen Londsdale Institute for Human Health Research, Maynooth University, W23 F2H6 Maynooth, Ireland

**Keywords:** copper(II) complexes, biotin, anticancer activity, 3D cell cultures

## Abstract

The interest in the use of copper as a metal scaffold for the development of novel chemotherapeutics has considerably grown in recent years. This is mainly due to the relatively lower toxicity of copper complexes with respect to platinum drugs (i.e., cisplatin), the different mechanisms of action, and the cheaper cost. In the last decades, hundreds of copper-based complexes were developed and screened as anticancer agents, with the antesignanus of all compounds being copper bis-phenanthroline [Cu(phen)_2_]^2+^ developed by D.S. Sigman in the late 1990s. In particular, copper(phen) derivatives have been shown high interest in their capacity to interact with DNA by nucleobase intercalation. Here, we report the synthesis and chemical characterization of four novel copper(II) complexes functionalised with phenanthroline derivatives containing biotin. Biotin, also known as Vitamin B7, is involved in a series of metabolic processes, and its receptors are often overexpressed in many tumour cells. A detailed biological analysis including cytotoxicity in 2D and 3D, cellular drug uptake, DNA interaction, and morphological studies are discussed.

## 1. Introduction

Platinum(II) anticancer drugs, cisplatin, carboplatin, and oxaliplatin have displayed phenomenal clinical activity in the treatment of solid tumours in the past several decades, and are used in nearly 50% of all clinical chemotherapeutic regimens [1,2,3]. Platinum-derived anticancer agents deliver their cytotoxicity mainly through covalent binding to the therapeutic target DNA rather than noncovalent interaction, a behaviour that is different from most of the classical nonmetal organic drugs, therefore resulting in enhanced anticancer activity due to the amplified duration of drug action [4,5,6]. The drawbacks associated with the use of these drugs are the offsite interactions between the platinum and biomolecules (such as proteins, enzymes…), which not only lead to chronic side effects but also to decreased drug efficacy. This effect, coupled with the increasing resistance developed by cancers to platinum complexes, opens the door for the need of designing new and highly selective-based anticancer therapeutics [1]. One strategy is to exploit the overexpression of specific receptors on or within the tumour cell. The use of specific vectors that can be recognized by these overexpressed receptors is a smart strategy to increase the selectivity of metal-based drugs enhancing the accumulation inside cancer tissues [7]. This strategy, paired with the use of an endogenous metal centre like copper(II), should lead to a more selective and less-toxic alternative to a platinum-based therapeutic arsenal [8].

**Figure 1:**
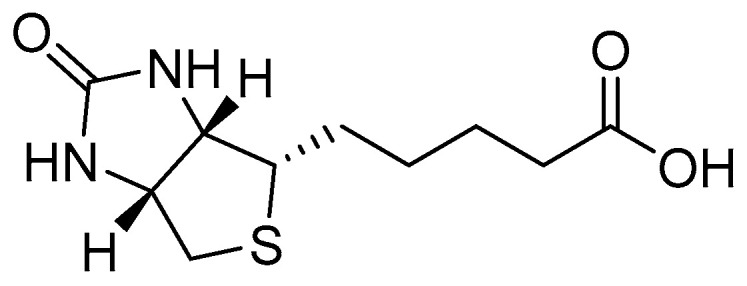
Structure of biotin.

Biotin belongs to the family of B vitamins, and it is more specifically known as vitamin B7 or vitamin H. Biotin is involved in a huge array of metabolic processes in mammals but also in many other organisms. Mostly, it is used for the regulation of carbohydrates and amino acids [9]. Biotin is a water-soluble heterocyclic molecule formed of two condensed five-member rings (a tetrahydrothiophene ring fused to an ureido ring) and a carboxylic acid at the end of a four-carbon chain which makes it a highly attractive delivery vector (Figure 1). Recent reports have shown that the biotin-specific uptake systems are enhanced in many cancer cells, including leukaemia (L1210FR), ovarian (Ov2008, ID8), colon (Colo-26), mastocytoma (P815), lung (M109), renal (RENCA, RD0995) and breast (4T1, JC, MMT06056) cancer cell lines [10]. Biotin has been demonstrated to be very rapidly taken up into tumour cells which overexpress the biotin-related receptors on their cell surface. Studies have already shown that tagging classical metal-based chemotherapy drugs with a biotin moiety leads to very positive effects [11]. Guo et al. developed several biotinylated Pt(IV) complexes with indomethacin [12,13] and, in 2017, Wang et al. created a library of Pt(IV) complexes with biotin in an axial position [14]. Very recently, in 2022, Han et al. developed a Pt(IV) complex bearing one HDAC inhibitor (4-phenylbutyric acid) and biotin moiety attached in the axial positions [15]. In 2019, Upadhyay et al. reported the synthesis and biological evaluation of platinum(II) complexes where a biotin moiety was conjugated to a 1,10-phenanthroline scaffold [16] and biotinylated ruthenium, osmium, and copper complexes are described [17,18,19,20,21].

**Scheme 1:**
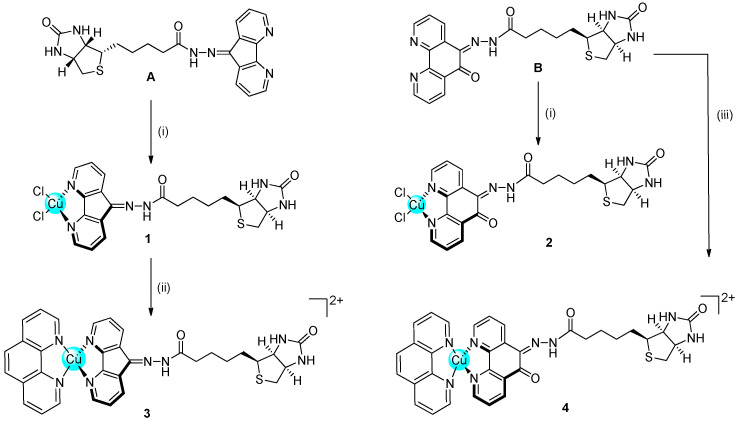
Synthetic pathway for the formation of complexes **1**–**4**. (i)—CuCl_2_/MeOH/reflux; (ii)—1,10-Phenanthroline/AgNO_3_/DMF/r.t; (iii)—[Cu(phen)(ONO_2_)_2_]/DMF/40 °C.

Inspired by these results, we here report the synthesis, characterization, and biological application of four novel Cu(II) complexes (**1**–**4**) with two different biotin-functionalized ligands **A** and **B** (Scheme 1). The addition of the biotin to the scaffolds of phenanthroline and dafone (4,5-Diazafluoren-9-one) will enhance not only the selectivity toward tumour tissues but also the hydrophilic features of the final complexes, which is an important characteristic for biological applications. The in vitro anticancer potential of these complexes has been investigated on 2D and 3D cancer-cell models; additionally, cell-free and in-cell studies were carried out in order to elucidate their mechanism of action.

## 2. Results and Discussion

### 2.1. Synthesis and Characterisation

Phenanthroline derivatives of Cu(II) have been historically studied as antimicrobial and anticancer compounds, with a strong ability to intercalate between the DNA nucleobases [22,23]. On these bases, we decided to modify the phenanthroline scaffold to allow functionalisation with the vector biotin. Two novel ligands (**A** and **B**) were conjugated to the Cu(II) centre, obtaining four novel complexes, two with two chlorides (**1** and **2**) and two with another phenanthroline (**3** and **4**) completing the copper coordination sphere (Scheme 1).

As described in the experimental section and in Scheme S1 (Supporting Information), biotin was transformed in the corresponding methyl ester derivative (**1**) before being functionalised with hydrazine to obtain the biotin-hydrazine derivative (**2**). Biotin-hydrazine **2** was then reacted with dafone (4,5-Diazafluoren-9-one) or phendione, obtaining the two novel ligands (**A** and **B**, respectively). The novel ligands were characterised by multinuclear NMR, IR, elemental analyses, and HR-MS (Figures S1–S6 in Supporting Information).

The synthesis of the four novel complexes **1**–**4** has been challenging and only after different attempts, the complexes were obtained in high purity for biological studies. Complexes **1** and **2** were obtained by reacting CuCl_2_ and the corresponding ligand (**A** or **B**) in MeOH. Complex **3** was obtained from complex **1** by reaction with one equivalent of phenanthroline in the presence of AgNO_3_. Complex **4**, finally, was obtained by direct reaction of ligand **B** with one equivalent of [Cu(phen)(ONO_2_)_2_] in DMF. All the complexes have been characterised by IR and HR-MS and the purity was assessed by elemental analyses and HPLC (Figures S7–S13 in Supporting Information). The complexes are stable in physiological buffer solutions in the pH range of 4–9 and are stable at pH 6 for at least one week (UV-Studies, Figure S14 in Supporting Information).

### 2.2. Cytotoxicity

The in vitro anticancer potential of the newly developed complexes and of the ligands has been tested on six different cancer cell lines including PSN-1 (pancreatic), HCT-15 (colorectal with low sensitivity for cisplatin), 2008 (ovarian), A431 (cervical), MDA-MB-231 (breast), and U1285 (lung) cancer cells. For comparison purposes, cisplatin efficacy was assessed under the same experimental conditions. The cytotoxicity parameters, expressed in terms of IC_50_ and obtained after 72 h of drug exposure by MTT assay, are reported in Table 1.

All the newly developed copper complexes showed strong cytotoxicity against most of the cell lines, with average IC_50_ values from three to fourteen times lower with respect to those elicited by the reference drug cisplatin. Ligands **A** and **B** did not show activity comparable to the complexes. Compound **4** was the most active complex in 2D assays, with an average IC_50_ value in the low micromolar range (0.8 μM) while the weakest was complex **1** (average IC_50_ 3.4 μM). Due to note, all the complexes were very effective in the MDA-MB-231 cell line, which overexpress the SMVT (sodium-dependent multivitamin transporter) receptor, which is well known to be involved in the intracellular translocation of biotin [24].

Table 2 reported the IC_50_ values of the complexes against two types of ovarian cancer cells, one sensitive (2008) and one resistant to cisplatin (C13*). Very interesting, all complexes showed similar activity in both cell lines, indicating that they can overcome the cisplatin resistance. The resistance factor (R.F., the ratio among IC_50_ values obtained in resistant C13*cells and those calculated in the sensitive 2008 ones) calculated for all tested complexes resulted to be ≤2 (R.F. for cisplatin 11.1), strongly confirming the ability of the complexes to bypass cisplatin resistance.

In order to preliminarily assess the possible toxic effect on noncancerous cells, we also evaluated the cytotoxic potential of the newly developed Cu(II) complexes against noncancerous CHO cells (Table 3). For all tested complexes, calculated IC_50_ values were higher than those obtained (on average) against tumour cells, thus indicating for this class of metal complexes a slightly preferential cytotoxicity against cancer cells, as attested by the calculated selectivity index values (SI = the quotient of the average IC_50_ toward normal cells divided by the average IC_50_ for the malignant cells).

The most active complexes, **2** and **4**, were also screened against 3D spheroids of human pancreatic PSN-1 cancer cells to further assess their anticancer potential in a more predictive environment. The 2D cell cultures reflect only in part the morphology of human cancer cells and do not represent the complexity of the in vivo tumour microenvironment regarding morphology, growth, gene expression, and differentiation. Conversely, the 3D cell cultures (spheroids), constitute an alternative and/or parallel approach to 2D, as they more accurately mimic the tumour microenvironment, and, nowadays, they are increasingly used as preclinical tumour models in anticancer drug screenings.

The cancer spheroids were treated with tested complexes for 72 h, and the cell viability was estimated by means of a modified acid phosphatase (APH) assay. The IC_50_ values for complexes **2** and **4** in 3D PSN-1 human pancreatic adenocarcinoma-cell spheroids are shown in Table 4. Both complexes proved to be far more active than cisplatin in 3D spheroids, being about forty and eight times more effective than the reference metallodrug, respectively. On the other hand, compared with 2D assays, complex **2** was found more cytotoxic than complex **4** in 3D studies.

### 2.3. Cellular Uptake

**Figure 2:**
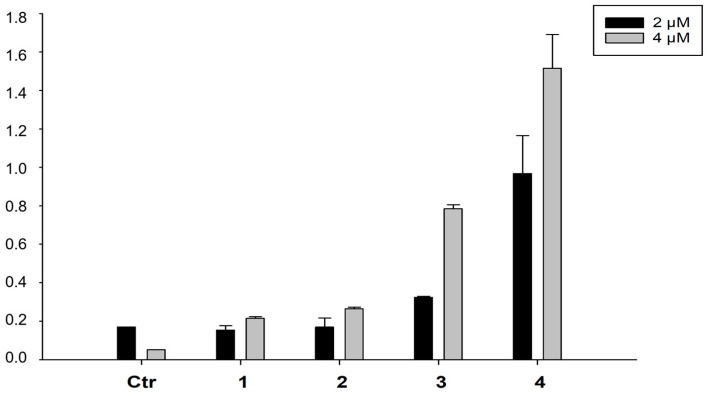
Cellular uptake of complexes **1**–**4** in drug-treated MDA-MB-231 human breast cancer cells. Cells were incubated for 24 h with 2 or 4 µM of tested complexes. The amount of cellular Cu was estimated by GF-AAS. Error bars are S.D.

The ability to enter cancer cells is a very important factor to determine the biological activity of metal-based compounds. Consequently, with the aim of correlating the antiproliferative activity elicited by all the newly developed complexes and their accumulation profiles into cancer cells, the Cu content was evaluated in the SMVT overexpressing MDA-MB-231 cells. Uptake profiles were determined by treating cancer cells for 24 h with two concentrations (2 and 3 μM) of tested complexes. Results, reported in Figure 2, clearly showed that the Cu intracellular content was dose dependent and, among all, complexes which are most internalised into cancer cells are derivatives **3** and **4**. These results suggest that the role of the biotin in the cellular uptake is minimal and that complexes are mainly internalised by passive diffusion, being that complexes **3** and **4** are the most lipophilic ones (due to the presence of phenanthroline with respect to the chlorides in complexes **1** and **2**). Interestingly, a linear and direct correlation between drug uptake and 2D cytotoxicity could be drawn for all tested complexes (Figure S15 in Supporting Information).

### 2.4. DNA Interaction and PDI Inhibition

**Figure 3:**
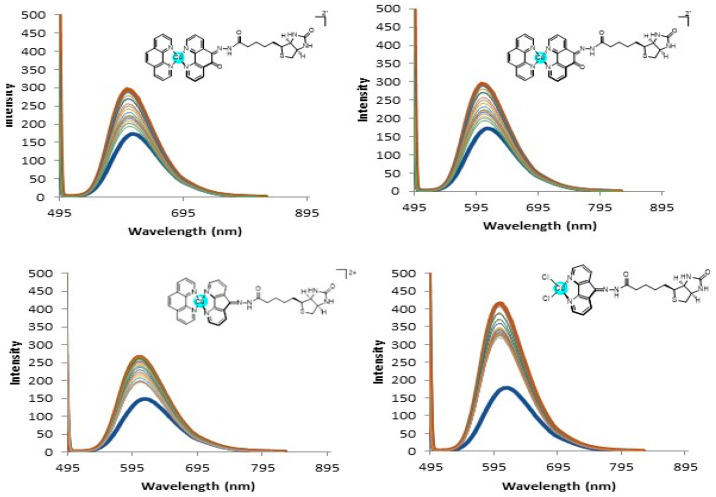
DNA Fluorescence quenching and relative displacement of EtBr for complexes **1**–**4**.

A number of research studies were performed on the elucidation of the mechanism of action of copper complexes in the past three decades. Despite several different molecular targets having been proposed for copper(II) complexes, DNA still represents one of the most substantiated when considering diamine ligands. On this basis, we thought it of interest to evaluate the ability of tested complexes to interact with DNA, both at molecular and cellular levels. An ethidium bromide (EB) displacement assay was used to understand if the complexes interact with the DNA at a molecular level. Compounds **3** and **4** displayed the highest quenching values and the relative fluorescence decrease was greater than 50% with binding constants of 2.53 × 10^7^ M(bp^−1^) and 1.28 × 10^7^ M(bp^−1^) for compounds **3** and **4**, respectively (Figure 3). These values are very much in line with what was previously seen by us [25] and also very close to the reported literature values of 2.60 × 10^7^ M(bp^−1^) and 3.04 × 10^7^ M(bp^−1^) for the bis complexes of [Cu(phen)_2_]^2+^ and [Cu(DPQ)_2_]^2+^ reported by Molphy et al. [26]. Compounds **1** and **2** instead showed very small quenching and the binding constants were not determined. It is largely possible that the presence of the 1,10-phenanthroline scaffold on compounds **3** and **4** is contributing to the increased displacement of the EtBr (probably by intercalation) and leading to a more noticeable decrease in the fluorescence.

**Figure 4:**
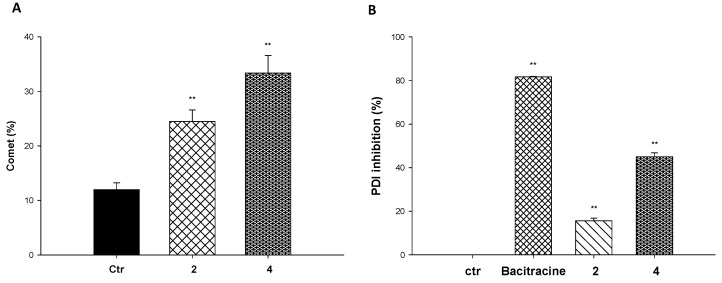
Mechanistic studies. (**A**) Comet assay: MDA-MDB-231 cells were treated for 3 h with 4 μM of tested compounds **2** and **4** or 200 µM of the reference drug chlorambucil. The relative percentage of comets (number of cells forming a comet/total number of cells) was detected in two randomly captured fields from two independent experiments. (**B**) PDI inhibition induced by tested compounds was measured by Proteostat PDI assay kit. The PDI inhibitor Bacitracine (0.5 mM) was used as a positive control. Error bars indicate S.D. ** *p* < 0.01 compared with control.

As proof of the principle of their DNA-targeting ability, the alkaline single-cell gel electrophoresis (Comet assay, Figure 4) assay was performed to evaluate cellular genomic DNA lesions induced by tested complexes. The most effective cytotoxic derivatives, complexes **2** and **4,** were chosen as the most representative compounds. MDA-MDB-231 cells were exposed for 3 h to 4 μM of tested compounds or 200 µM of the reference drug chlorambucil. Figure 4A depicts the relative percentage of comets and the number of cells forming a comet relative to the total number of detected cells per each condition in two randomly captured fields from two independent experiments. Comet assay studies clearly showed that at least one fourth and one third of cells treated with compounds **2** and **4**, respectively, presented lesioned genomic DNA. Interestingly, the DNA damaging efficacy of compound **4** was even much higher than that induced by the reference compound chlorambucil. On the contrary, cell metalation studies clearly showed that complexes are not able to covalently bind the DNA (Figure S16 in Supporting Information). Altogether, these data confirm that DNA is a major target for this class of complexes and the interaction is mainly by intercalation. On the other hand, some reports highlighted protein disulphide isomerase (PDI) as an emerging target for copper complexes [27]. On this basis, we also evaluated the ability of our complexes to act as PDI inhibitors. The enzyme was treated with 25 µM of complexes **2** and **4**, and the ability to hamper its activity was assessed by a biochemical colourimetric method (Proteostat kit). As shown in Figure 4B, both complexes are able to inhibit the PDI by 45 and 15%, respectively, indicating that PDI could be a possible secondary molecular target for this class of copper(II) complexes.

**Figure 5:**
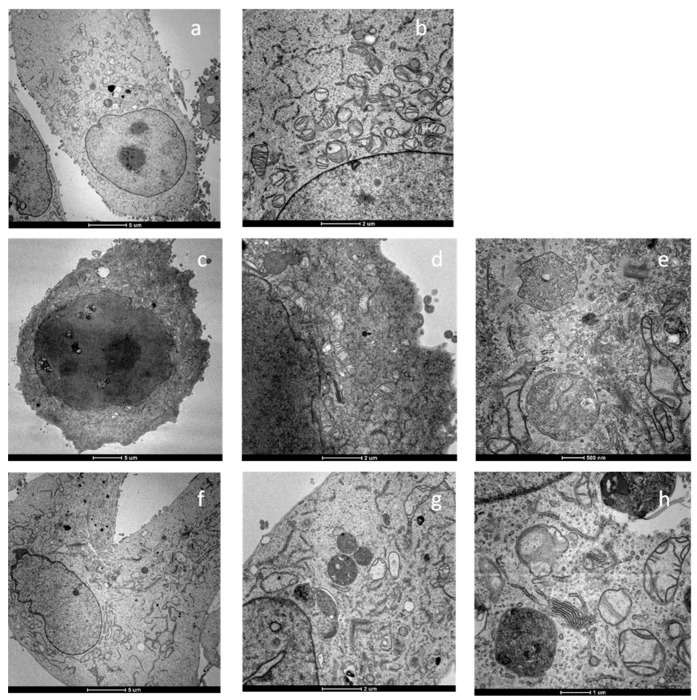
TEM analysis of MDA-MB-231 human breast cancer cells: (**a**,**b**) control cells; (**c**–**e**) cells treated for 24 h with IC_50_ concentrations of **2**; (**f**–**h**) cells treated for 24 h with IC_50_ concentrations of **4**.

Transmission electron microscopy (TEM) studies were also performed to assess the eventual modification of cellular/subcellular morphological features induced by the tested complexes. Figure 5 reports the TEM images of the breast carcinoma cells MDA-MB-231 treated for 24 h with IC_50_ concentrations of cisplatin or compounds **2** and **4**. Cells treated with compounds **2** and **4** showed a very different morphology with respect to the untreated cells and were characterized by a substantial enhancement of the mitochondrial dimensions (*swelling*) and a partial disruption of the cristae structures. Furthermore, an extensive increase in cytoplasmatic multivesicular bodies and in nuclear multilamellar bodies was detected. It is reported that the multivesicular bodies derive from activation of the lysosomes and, therefore, this suggests that the mechanism of action involves cytoplasmic acidic organelles.

### 2.5. Apoptosis and Morphological Studies

**Figure 6:**
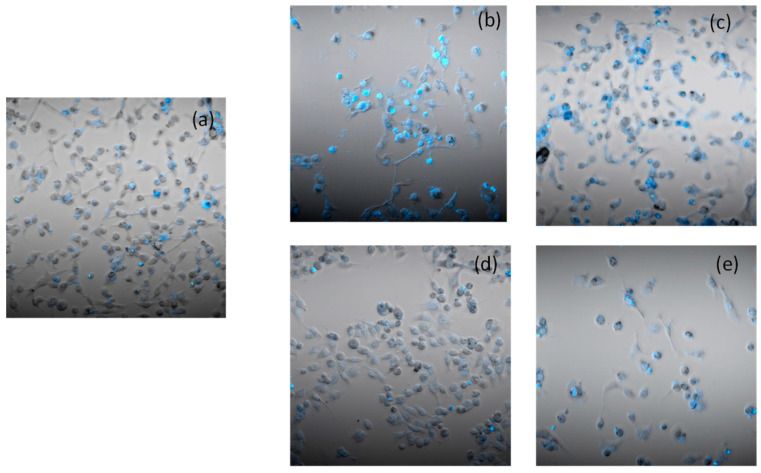
Hoechst staining of MDA-MB-231 cells: (**a**) control cells; cells incubated for 24 h (**b**) or 48 h (**c**) with IC_50_ of cisplatin; cells incubated for 24 h (**d**) or 48 h (**e**) with IC_50_ of **4**.

In order to assess the ability of the most representative complex **4** to induce cancer cell death by apoptosis, the Hoechst33258 assay was performed on human breast carcinoma cells MDA-MB-231. Cells were incubated with complex **4** at 3 μM for 24 and 48 h. Cells treated with derivative **4**, similar to cisplatin, presented brightly stained nuclei and morphological features typical of cells undergoing apoptosis, such as chromatin condensation and fragmentation (Figure 6) in a time-dependent manner. These results clearly indicate that the newly developed copper(II) complexes induced cancer-cell death by apoptosis.

## 3. Experimental Part

### 3.1. Material and Methods

All reactants and reagents were purchased from commercial sources, in particular Sigma-Aldrich and Fluorochem. All solvents were used without further purification. Dafone [28], phendione [25] and [Cu(phen)(ONO_2_)_2_] [29] were synthesised as previously reported with minor modifications.

The description of the instruments and of the experiments to assess the purity of the samples via HPLC are described in previous papers [25,30,31,32].

#### 3.1.1. Stability

The pH stability was examined over a pH range of 4–9 by preparing a 10 µM solution of each compound in H_2_O and adjusting the pH using 0.01 mL of either NaOH 1 M or 0.1 M and HCl of the same in order to keep the volume consistent. When the desired pH was reached, the solution was allowed to stabilise for 10 min at 18 °C before it was then examined in a UV-Vis spectrometer at over a range of 200–600 nM. The stability at pH = 6 was assessed over a period of 1 week.

#### 3.1.2. Ethidium Bromide Displacement Assay

EtBr displacement assays were performed in 10 mM phosphate buffer solution (pH 7.4) as described by Boger et al. [33] The binding values were calculated using the % relative decrease of fluorescence at 50% and the equation below.

(1) $$E_{EB}\frac{\left[EtBr\right]}{\left[compound\right]50{\%}_{FI}}$$

#### 3.1.3. Experiments with Cultured Human Cancer Cells

Tested compounds were dissolved in DMSO and added to the cell-growth medium to a final solvent concentration of 0.5%, which is well reported to not have cytotoxic effects on cell viability. Cisplatin (Sigma Chemical Co., St. Louis, MO, USA) was dissolved in a physiologic solution (0.9% sodium chloride).

#### 3.1.4. Cell Cultures

Human colon (HCT-15), pancreatic (PSN-1), and breast (MDA-MB-231) carcinoma-cell lines were obtained from the American Type Culture Collection (ATCC, Rockville, MD). Human cervical carcinoma (A431) cells were kindly provided by Prof. F. Zunino (Division of Experimental Oncology B, Istituto Nazionale dei Tumori, Milan). Human ovarian carcinoma (2008) cells and their cisplatin-resistant counterpart (C13*) were kindly provided by Prof. G. Marverti (Dipartimento di Scienze Biomediche, Università di Modena University, Italy). Cell lines were cultured in RPMI-1640 or DMEM medium (Euroclone) added with 10% fetal calf serum (Euroclone, Milan, Italy), antibiotics (50 units per mL penicillin, and 50 μg mL^−1^ streptomycin), and 2 mM L-glutamine.

#### 3.1.5. MTT Test

The growth inhibitory effect toward the tumour cell lines was evaluated by the MTT test as previously described [34].

#### 3.1.6. Spheroid Cultures

Spheroid cultures were performed by seeding 2.5 × 10^3^ PSN-1 cancer cells/well in spheroid-suitable tissue culture untreated 96-well plates (Greiner Bio-one, Kremsmünster, Austria) in RPMI-1640 cell culture medium without phenol red (Sigma Chemical Co., St. Louis, MO, USA) containing 10% fetal calf serum and supplemented with 20% methyl cellulose stock solution.

#### 3.1.7. APH Assay

An established APH-modified assay was used to detect the cytotoxicity profile in 3D spheroids, as previously described. IC_50_ values were calculated using a 4-PL model [35,36].

#### 3.1.8. Copper Cellular Content

MDA-MB-231 cells (2.5 × 10^6^) were seeded in 75 cm^2^ flasks in a growth medium (20 mL). Following 24 h, cells were treated for 24 h with tested complexes and samples processed as previously described [27].

#### 3.1.9. Comet Assay

About 3 × 10^4^ MDA-MB-231 cells were seeded in 25 cm^2^ flasks in a growth medium. After overnight incubation, cells were treated for 3 h with 2.5 μM of tested compounds and processed as previously described [27]. Pictures were captured with a Zeiss LSM-800 confocal microscope (Zeiss, Oberkochen, Germany). All photos were analyzed by the Zen 2.3 system (Zeiss, Oberkochen, Germany).

#### 3.1.10. Protein Disulfide Isomerase (PDI) Activity

The reductase activity of PDI was assayed by measuring the PDI-catalysed reduction of insulin in the presence of increasing concentrations of the tested compounds by using the PROTEOSTAT PDI assay kit (Enzo Life Sciences, Lausen, Switzerland). Experiments were performed according to the manufacturer’s instructions as previously described [27]. IC_50_ values were calculated by the 4-PL model.

#### 3.1.11. Transmission Electron Microscopy (TEM) Analyses

About 10^6^ MDA-MB-231 cells were seeded in 24-well plates and, following overnight incubation, were treated with the tested compounds for 24 h. Then, cells were processed as previously described [1]. Pictures were captured with a Hitachi H-600 electron microscope (Hitachi, Tokyo, Japan) operating at 75 kV. All photos were analyzed by using Corel Draw 11.

#### 3.1.12. Cell Death Induction

MDA-MB-231 cells were seeded into 8-well tissue-culture slides (BD Falcon, Bedford, MA, USA) at 5 × 10^4^ cells/well (0.8 cm^2^). After overnight incubation, cells were treated with IC_50_ doses of the tested compound and processed as previously described [27].

#### 3.1.13. Statistical Analysis

All values are the means ± SD of no less than three measurements starting from three different cell cultures. Multiple comparisons were made by ANOVA followed by the Tukey–Kramer multiple comparison test (* *p* < 0.05, ** *p* < 0.01), using GraphPad InStat software 3.10 (GraphPad Software, San Diego, CA, USA).

### 3.2. Synthesis and Characterisation

The ligands **A** and **B** were synthesised according to Scheme S1 in Supporting Information.

#### 3.2.1. 1,10-Phenanthroline-5,6-dione (phendione)

The phendione was prepared using a previous method from our group [24]; 1,10-phenanthroline (2000 mg, 10 mmoles) and potassium bromide (2000 mg, 16.8 mmoles) were added to a round bottom flask containing an ice-cold mixture of H_2_SO_4_ (20 mL) and HNO_3_ (10 mL). The solution was refluxed at 100 °C for 3 h and a dark-orange colour was observed. The solution was allowed to cool to room temperature, then slowly poured into deionised iced H_2_O (200 mL). To this was added slowly a solution of NaOH (200 mL, 5 M) until a pH of ~6 was reached. The solution was then poured into a separation funnel and washed with CHCl_3_ (2 × 50 mL). The organic phase was dried using anhydrous sodium sulphate. The solution was then filtered and reduced in vacuo. A yellow solid was recovered. The crude yellow solid was purified via recrystallization from MeOH to give yellow needles (162 mg, 77%). ^1^H NMR (500 MHz, CDCl_3_) *δ* 9.11 (dd, 2H), 8.49 (dd, 2H), 7.58 (dd, *J* = 7.9, 4.7 Hz, 2H). ^13^C NMR (126 MHz, CDCl_3_) *δ* 178.8, 156.5, 153.0, 137.4, 128.2, 125.7. Elemental Analysis: C_12_H_6_N_2_O% Calculated C 68.57, H 2.88, N 13.51, % Found: C 67.97, H 3.07, N 13.14 HR-MS (+): *m*/*z* Calculated for C_12_H_6_N_2_O + [H^+^] 211.0508, Found 211.0507.

#### 3.2.2. Dafone (4,5-Diazafluoren-9-one)

Dafone was synthesised as reported in the literature with modifications [28]; 1,10-phenanthroline (1000 mg, 5.54 mmoles) and KOH (1010 g, 18.1 mmoles) were refluxed into H_2_O (60 mL). In a separate flask KMnO_4_ (2540 mg, 16.01 mmoles) was dissolved in warm H_2_O (35 mL, 85 °C). The KMnO_4_ was then added dropwise with stirring over the course of 3.5 h. It was kept at 85 °C for the duration of the addition. After the final addition, the stirring was allowed to continue for 1 h. The brown solution was gravity filtered while hot and the brown solid was washed extensively with H_2_O. The orange filtrate was allowed to cool to room temperature and then extracted with CHCl_3_ (3 × 150 mL). The organic layers were combined and washed with brine (100 mL), then dried over Na_2_SO_4_ anhydrous. The solvent was removed under reduced pressure to yield the crude yellow/orange solid. The crude product was purified via recrystallization from acetone to give needles (450 mg, 45%). ^1^H NMR (500 MHz, CDCl_3_) δ 8.80 (ddd, *J* = 5.0, 1.5, 1.0, 2H), 7.99 (ddd, *J* = 7.5, 1.5, 1.1, 2H), 7.35 (ddd, *J* = 7.5, 5.0, 0.8, 2H). ^13^C NMR (126 MHz, CDCl_3_) δ 189.7, 163.5, 155.3, 131.6, 129.4, 124.8. Elemental Analysis: C_11_H_6_N_2_O % Calculated: C 72.52, H 3.32, N 15.38. % Found: C 73.06, H 3.83, N 15.94. HR-MS (+): *m*/*z* Calculated for C_11_H_6_N_2_O + [ H^+^] 183.0482, Found 183.0561.

#### 3.2.3. Biotin Methyl Ester (**1**)

The synthesis of biotin methyl ester was performed according to a modified protocol previously reported [37]. Biotin (300 mg, 1.23 mmoles) was suspended in dry MeOH (3 mL) and placed under N_2_. Following this, the system was flushed entirely with N_2_. SOCl_2_ (0.3 mL, 4 mmoles) was then added dropwise via cannula. The solution was allowed to stir overnight at room temperature. A clear golden solution was observed. The excess SOCl_2_ and MeOH were removed under reduced pressure. The flask was cooled in an ice bath for an hour and a crystalline solid was obtained. The white solid was taken up in CHCl_3_ (25 mL) and washed with NaHCO_3_ (2 × 30 mL) and then with brine (25 mL). The organic layer was dried over Na_2_SO_4_ (anhydrous) and the solvent was removed under reduced pressure to yield the title compound as a white solid (269 mg, 85%). ^1^H NMR (500 MHz, DMSO-*d*_6_) *δ* 6.46 (s, 1H), 6.38 (s, 1H), 4.30 (dd, *J* = 7.5, 5.3, 1H), 4.13 (m, 1H), 3.57 (s, 3H, O-C*H*_3_), 3.09 (m, 1H), 2.81 (dd, *J* = 12.4, 5.1, 1H), 2.56 (t, *J* = 13.4, 1H), 2.29 (m, 2H), 1.53 (m, 4H), 1.27 (m, 2H). ^13^C NMR (126 MHz, DMSO-*d*_6_) *δ* 173.3162.7, 61.1, 59.3, 55.4, 51.2, 48.6, 33.1, 28.1, 28.0, 24.5. Elemental Analysis: C_11_H_18_N_2_0_3_S % Calculated: C 51.14, H 7.02, N 10.84% Found: C 50.86, H 6.77, N 10.56.

#### 3.2.4. Biotin Hydrazide (**2**)

Biotin-hydrazide was synthesised as reported in the literature with slight modification [38]. Biotin methyl ester (200 mg, 0.77 mmoles) was suspended in dry MeOH (10 mL) and placed under N_2_. Hydrazine hydrate (210 mg, 6.77 mmoles) was added dropwise via cannula to the suspension of biotin methyl ester. The solution was warmed to 50 °C until a clear solution was observed (10–12 min). The solution was allowed to stir for 24 h at room temperature and a white precipitate was observed. The MeOH was removed under reduced pressure to yield a white solid. The crude product was taken up in warm H_2_O (40 mL) and the aqueous layer was washed with CHCl_3_ (3 × 30 mL). The aqueous layers were combined, and the H_2_O was removed under reduced pressure to yield a white solid (184 mg, 92%). R_f_ 0.72 (DCM: MeOH 90:10). ^1^H NMR (500 MHz, DMSO-*d_6_*) *δ* = 8.93 (s, 1H, *N*-*H*), 6.44 (s, 1H), 6.37 (s, 1H), 4.30 (dd, 1H), 4.19 (s, 2H, N-*H*_2_), 4.12 (m, 1H), 3.09 (m, 1H), 2.82 (dd, *J* = 12.4, 5.1, 1H), 2.61 (m, 1H), 2.00 (m, 2H), 1.43 (m, 6H). ^13^C NMR (126 MHz, DMSO-*d_6_*) *δ* = 175.6, 165.3, 62.0, 60.2, 55.2, 39.6, 33.3, 27.7, 27.5, 24.8. Elemental Analysis: C_10_H_18_N_4_0_2_S % Calculated: C 46.49, H 7.02, N 21.69% Found: C 46.19, H 7.16, N 20.46. HR-MS (+): *m*/*z* Calculated for C_10_H_18_N_4_O_2_S + [Na^+^] 281.1047, Found 281.1049.

#### 3.2.5. Biotin-Dafone (**A**)

Biotin hydrazide (138 mg, 0.537 mmoles), dafone (98 mg, 0.537 mmoles), and *p*-TSA (*p*-Toluenesulfonic acid 10% mol) were suspended in EtOH (20 mL). The solution was refluxed until a clear solution was observed (1–1.5 h). The solution was then allowed to stir for 24 h at room temperature. A white precipitate was observed. The white solid was collected via Buchner filtration, washed with CHCl_3_ (2 × 30 mL) and dried under a high vacuum. No further purification was required (112 mg, 54%). ^1^H NMR (500 MHz, DMSO-*d_6_*) δ 11.38 (s, 1H, *H*-N-N), 8.73 (dd, *J* = 11.9, 7.0, 3H, Ar-H), 8.22 (s, 1H, Ar-H), 7.51 (m, 2H, Ar-H), 6.47 (d, *J* = 4.7, 1H, C=O-N-*H*), 6.38 (d, *J* = 5.2, 1H, C=O-N-*H*), 4.32 (d, *J* = 4.4, 1H, NC*H*), 4.12 (m, 2H, S-C*H_2_*), 3.17 (m, 2H, SC*H* & NC*H*), 2.83 (d, broad, *J* = 5.0, 2H, C=O-C*H_2_*), 1.78–1.28 (m, 6H, 3 × C*H_2_*). ^13^C NMR (126 MHz, DMSO-*d_6_*) δ 162.7, 158.4, 156.7, 151.3, 140.4, 134.5, 132.4, 129.0, 124.3, 123.5, 89.8, 87.7, 61.1, 59.2, 55.4, 48.6, 40.4 (under DMSO, DEPT—135), 28.3, 28.1, 24.5, 16.9. Elemental Analysis: C_21_H_22_N_6_O_2_S% Calculated: C 59.70, H 5.25, N 19.89, % Found: C 59.06, H 5.16, N 19.46. HR-MS (+): *m*/*z* Calculated for C_21_H_22_N_6_O_2_S + [Na^+^] 445.1423, Found 445.1390.

#### 3.2.6. Biotin-Phen (**B**)

Biotin hydrazide (245 mg, 0.948 mmol) was suspended in warm EtOH (20 mL, 50 °C). In a separate flask, phendione (200 mg, 0.948 mmol) was dissolved in warm EtOH (20 mL, 50 °C). The phendione was then added dropwise while stirring to the biotin hydrazine over the course of 45 min. A yellow mixture was observed. *P*-TSA (10% mol) was added and after 20 min the solution turned a clear-yellow colour. The solution was allowed to reflux for 12 h and then allowed to cool to room temperature. The EtOH was reduced under vacuum to 25% volume and the flask was placed in the fridge overnight. A bright-yellow precipitate was observed. The yellow solid was taken up in CHCl_3_ (20 mL), washed with brine (2 × 30 mL), and dried over Na_2_SO_4_. The excess CHCl_3_ was removed under reduced pressure to yield a bright-yellow solid. The crude product was purified via silica-gel chromatography (95:5) DCM/MeOH (360 mg, 85%). R_f_ 0.88. ^1^H NMR (500 MHz, DMSO-*d_6_*) *δ* = 13.97 (s, 1H, N-*H*), 9.08 (dd, *J* = 4.5, 1.8, 1H, Ar-H), 8.86 (dd, *J* = 4.5, 1.7, 1H, Ar-H), 8.55 (m, *J* = 8.0, 1.7, 2H, Ar-H), 7.73 (dd, 1H, Ar-H), 7.64 (dd, *J* = 8.1, 4.5, 1H, Ar-H). 6.48 (s, 1H, CN-*H*), 6.37 (s, 1H, CN-*H*), 4.31 (dd, *J* = 7.6, 5.2, 1H, C*H*), 4.15 (m, 1H, C*H*), 3.14 (m, 2H, SC*H*_2_), 2.83 (m, 2H, amide C*H*_2_), 2.60 (m, 1H, SC-*H*), 1.71–1.52 (m, 6H, 3× C*H*_2_). ^13^C NMR (126 MHz, DMSO-*d_6_*) *δ* = 180.8, 162.7, 155.5, 152.2, 150.6, 146.2, 135.7, 131.5, 131.4, 130.8, 128.4, 127.4, 125.0, 124.0, 79.2, 61.0, 59.2, 55.4, 54.9, 40.1 (under DMSO dept—135), 28.1, 24.1. Elemental Analysis: C_22_H_22_N_6_O_3_S% Calculated: C 58.65, H 4.92, N 18.65, % Found: C 58.40, H 5.11, N 18.22. HRMS (ESI +): *m*/*z* Calculated for C_22_H_22_N_6_O_3_S + [Na^+^] 473.1372, Found 473.1366.

#### 3.2.7. [Cu(phen)(ONO_2_)_2_]

[Cu(phen)(ONO_2_)_2_] was synthesised as reported in the literature with slight modification [28]; 1,10-Phenanthroline (250 mg, 1.38 mmoles) was dissolved in methanol (30 mL). To this was added Copper (II) nitrate trihydrate (366 mg, 1.51 mmoles) dissolved in MeOH (5 mL). The solution was refluxed for 2 h at 80 °C. A bright-blue colour was observed. The solution was allowed to cool to room temperature. Et_2_O (20 mL) was added while stirring continued. A blue precipitate was observed. The precipitate was collected via vacuum filtration and washed with Et_2_O (20 mL). The blue solid was purified via recrystallization from MeOH to give bright-blue needles (225 mg, 44%). Elemental Analysis C_12_H_8_CuN_4_O_6_ + H_2_O% Calculated: C 37.36, H 2.61, N 14.52, % Found: C 37.81, H 2.96, N 14.21.

#### 3.2.8. [Cu(dafone-biotin)Cl_2_] (**1**)

A (153 mg, 0.363 mmol) was suspended in refluxing MeOH (40 mL). CuCl_2_.2H_2_O (68 mg, 0.398 mmol) was added dropwise while stirring. A green suspension was observed and after 5–6 min a clear solution was observed. The solution was allowed to reflux for another 4 h at which stage a fine-green precipitate was formed. The solution was allowed to stir for a further 3 h and then was cooled at r.t. and the green solid was collected via Buchner filtration and washed with cold MeOH (98 mg, 49%). Elemental Analysis: C_21_H_22_Cl_2_CuN_6_O_2_S% Calculated: C 45.29, H 3.98, N 15.09, % Found: C 45.88, H 3.48, N 14.80. IR (ATR): 3335, 2922, 1655, 1610, 1481, 1086, 741. cm^−1^. HPLC (RP): CH_3_CN: H_2_O (80:20) RT 2.17 min 100%. HR-MS (+): *m*/*z* Calculated for C_21_H_22_Cl_2_CuN_6_O_2_S [M]^+^ 555.0230, Found 555.0021.

#### 3.2.9. [Cu(phendione-biotin)Cl_2_] (**2**)

**B** (246 mg, 0.546 mmol) was suspended in MeOH (30 mL) and warmed to 50 °C and a solution of CuCl_2_.2H_2_O (111 mg, 0.655 mmol) in MeOH was added dropwise. The solution was refluxed. After 10–15 min a clear-green solution was observed for a brief few moments before a green precipitate began to form. The reflux was allowed to continue for a further 4 h before allowing the solution to cool to room temperature. The fine-green precipitate was collected via Buchner filtration and washed very gently with CHCl_3_ (10 mL) to dispel any unreacted ligand. No further purification was required (148 mg, 47%). Elemental Analysis: C_22_H_22_Cl_2_CuN_6_O_3_S% Calculated: C 45.17, H 3.79, N 14.37, % Found: C 45.78, H 3.91, N 14.76. IR (ATR): 3341, 2907, 1650, 1608, 1480, 1078, 741. cm^−1^. HPLC (RP): CH_3_CN: H_2_O (80:20) RT 1.97 min 100%. HR-MS (+): *m*/*z* Calculated for C_22_H_22_CuN_6_O_2_S [M^+^] 583.0147, Found 584.0004.

#### 3.2.10. [Cu(phen)(dafone-biotin)](NO_3_)_2_ (**3**)

One (104 mg, 0.180 mmol) was dissolved in anhydrous DMF (20 mL). AgNO_3_ (61 mg, 0.36mmol) dissolved in anhydrous DMF (3 mL) was added to the solution containing [Cu(dafone-biotin)Cl_2_] (**1**) and the solution was allowed to stir at r.t and in darkness for 4 h. An extremely fine white precipitate was observed. The white precipitate was filtered off and 2 drops of HCl (1M) were added to the filtrate to examine for excess Ag. The filtrate was filtered a second time and the clear-green filtrate was placed back into a round bottom and protected from light. To this was added solid 1,10-phenanthroline (35 mg, 0.0180 mmol). Immediately a deep-green solution was observed. The solution was allowed to stir overnight at r.t.; the DMF was reduced in vacuo and the resulting solution precipitated into excess Et_2_O. A light-green solid was observed. This solid was filtered and washed with CHCl_3_ (20 mL) and dried under a high vacuum (108 mg, 74%). HPLC (RP): CH_3_CN: H_2_O (80:20) RT 3.34 min 97% 3.85 min 3%. Elemental Analysis: C_33_H_30_CuN_10_O_8_S % Calculated: C 50.16, H 3.83, N 17.72. % Found: C 49.72, H 3.50, N 17.28.

#### 3.2.11. [Cu(phen)(phendione-biotin)](NO_3_)_2_ (**4**)

**B** (73 mg, 0.162 mmol) was dissolved in DMF (15 mL); in a separate flask [Cu(phen)(ONO_2_)_2_] (66 mg, 0.162 mmol) was dissolved in DMF (5 mL). The copper(II) solution was then added dropwise with stirring to the solution of biotin-phen **B**. A green colour was observed. The solution was allowed to stir for 24 h at 40 °C after which a brown colour was observed. The solution was allowed to cool to room temperature and diethyl—ether (30 mL) was added. A pale precipitate was observed. The brown solid was filtered off and the filtrate was concentrated in vacuo. The remaining green concentrate was precipitated into excess Et_2_O and the pale-green solid was collected. The solution was spun in the centrifuge for 10 min at 4000 rpm and a dark-green pellet was recovered. The pellet was washed gently with CHCl_3_ (82 mg, 67%). Elemental Analysis: C_34_H_30_CuN_8_O_9_S% Calculated: C 49.91, H 3.70, N 17.12,% Found: C 49.42, H 3.46, N 16.82. IR (ATR): 3058, 1647,1518, 1361, 1298, 1033, 846, 718. cm^−1^. HPLC (RP): CH_3_CN: H_2_O (80:20) RT, 3.23 min, 100%. HR-MS (+): *m*/*z* Calculated for C_34_H_30_CuN_6_O_3_S [M-2(NO_3_)]^+^ 693.1442, Found 693.1444.

## 4. Conclusions

In this research article, we have described the synthesis and the chemical characterisation of four novel copper(II) complexes functionalised with phenanthroline derivatives containing biotin. Biotin (Vitamin B7) plays a major role in many metabolic pathways and its receptors are overexpressed in many different cancer cell types. Two of the final complexes (**1** and **2**) contain two chlorides while the other two complexes (**3** and **4**) contain a phenanthroline ligand at the completion of the coordination sphere. The complexes were found stable in physiological conditions and over the pH range of 4–9. A detailed biological screening revealed that the four complexes possess strong anticancer properties against five different cancer cells, being also effective in overpassing cisplatin resistance in C13* ovarian cancer cells. The most promising complexes **2** and **4** showed strong activity also in 3D pancreatic cancer-cell cultures. The complexes are intracellularly uptaken probably by passive diffusion, as complexes **3** and **4** containing an extra phenanthroline moiety characterized by superior lipophilicity showed the highest internalisation profiles. Mechanistically, the principal molecular targets seem to be the protein disulphide isomerase (PDI) together with the nuclear DNA (electrostatic interaction rather than covalent DNA adducts). Morphological studies of the most promising complex 4 on SMVT overexpressing human breast carcinoma cells MDA-MB-231 indicate that the newly developed copper(II) complex elicited cell death by means of apoptosis. Unfortunately, the cellular-uptake studies do not clarify if biotin is playing the role of a targeting vector, but the lower cytotoxicity shown in normal cells with respect to the tumour cells is important to pursue for research in this field. Further biotinylating strategies will be taken into account in the development of novel biotin-functionalised metal complexes.

## Figures and Tables

**Table 1:** IC_50_ values of complexes **1**–**4** in PSN-1 (pancreatic), HCT-15 (colorectal with low sensitivity for cisplatin), 2008 (ovarian), A431 (cervical), MDA-MB-231 (breast), and U1285 (lung) cancer cells. Cells (3–5 × 10^3^ × well) were treated for 72 h with tested compounds. Cell viability was estimated by means of the MTT test. The IC_50_ values were calculated by a four-parameter (4-PL) logistic model (*p* < 0.05). S.D. = standard deviation.

Complex	PSN-1	HCT-15	2008	A431	MDA-MB-231	U-1285
**1**	0.6 ± 0.2	0.4 ± 0.1	4.5 ± 0.6	7.2 ± 0.6	2.1 ± 0.4	5.9 ± 0.5
**2**	0.4 ± 0.1	0.4 ± 0.1	1.1 ± 0.3	1.2 ± 0.5	1.4 ± 0.1	3.8 ± 0.5
**3**	0.7 ± 0.2	1.8 ± 0.2	3.3 ± 0.1	0.9 ± 0.2	1.4 ± 0.4	3.0 ± 0.3
**4**	0.6 ± 0.2	0.4 ± 0.02	0.6 ± 0.2	0.3 ± 0.03	0.6 ± 0.1	2.0 ± 0.6
**A**	>50	>50	>50	>50	>50	>50
**B**	N/A	N/A	21.48 ± 0.72	N/A	N/A	28.13 ± 2.77
**Cis** **platin**	12.1 ± 2.9	13.9 ± 1.7	2.2 ± 1.4	2.1 ± 0.9	30.5 ± 2.6	2.1 ± 0.9

**Table 2:** IC_50_ values of complexes **1**–**4** against 2008 and C13* ovarian cancer cell lines. Cells (3 × 10^3^ × well) were treated for 72 h with tested compounds. Cell viability was estimated by means of the MTT test. The IC_50_ values were calculated by a four-parameter (4-PL) logistic model (*p* < 0.05). S.D. = standard deviation. RF = IC_50_ resistant/IC_50_ parental cells.

Complex	2008	C13*	R.F.
**1**	4.5 ± 0.6	2.0 ± 0.8	0.4
**2**	1.1 ± 0.3	1.1 ± 0.4	1.0
**3**	3.3 ± 0.1	2.5 ± 0.2	0.7
**4**	0.6 ± 0.2	1.1 ± 0.3	1.9
**Cisplatin**	2.2 ± 1.4	24.1 ± 3.0	11.1

**Table 3:** Cytotoxic potential of the developed Cu(II) complexes against noncancerous CHO cells.

Complex	CHO	S.I.
**1**	13.1 ± 0.9	3.9
**2**	5.2 ± 0.3	3.7
**3**	2.1 ± 0.8	1.1
**4**	1.8 ± 0.7	2.3
**Cisplatin**	24.1 ± 3.0	11.1

**Table 4:** IC_50_ values of complexes **2** and **4** against human pancreatic PSN-1 cancer-cell spheroids. The cells (2.5 × 10^3^ cells/well) were treated for 72 h with tested compounds. Cell viability was estimated by means of the APH test. IC_50_ values were calculated from the dose-response curves by a 4-PL logistic model (*p* < 0.05). S.D. = standard deviation.

Complex	PSN-1
**2**	1.3 ± 0.5
**4**	6.9 ± 1.3
**Cisplatin**	52.6 ± 3.8

## Data Availability

The data presented in this study are available in the article and in the Supplementary Material.

## Supplementary Materials

The following supporting information can be downloaded at: https://www.mdpi.com/article/10.3390/molecules28104112/s1, Synthetic Schemes, NMR Spectra, HPLC Chromatograms, ESI-MS, UV-pH dependance and further biological assays.
