# Supplementary material for: Novel Biotinylated Cu(II)-Phenanthroline Complexes: 2D and 3D Cytotoxic Activity and Mechanistic Insight
Source: Molecules. 2023 May 16;28(10):4112. doi: 10.3390/molecules28104112 (PMC10222323; doi:10.3390/molecules28104112)
Supplement: Supplementary file 1 [file molecules-28-04112-s001.zip › molecules-2372026-supplementary.pdf]

Supplementary Information

Novel Biotinylated Cu(II)-Phenanthroline Complexes: 2D and 3D Cytotoxic Activity and Mechanistic Insight

Stephen Barrett, Michele De Franco, Chiara Donati, Cristina Marzano, Valentina Gandin and Diego Montagner

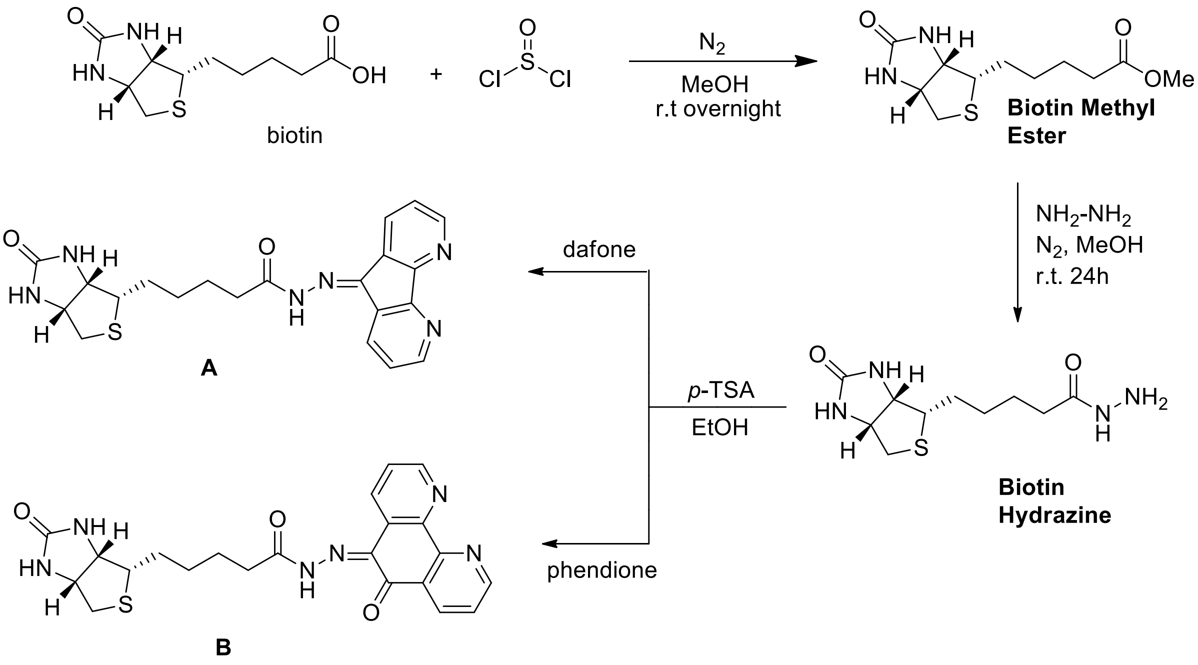

Scheme S1. Synthetic pathway for the production of the two biotinylated ligands **A** and **B**.

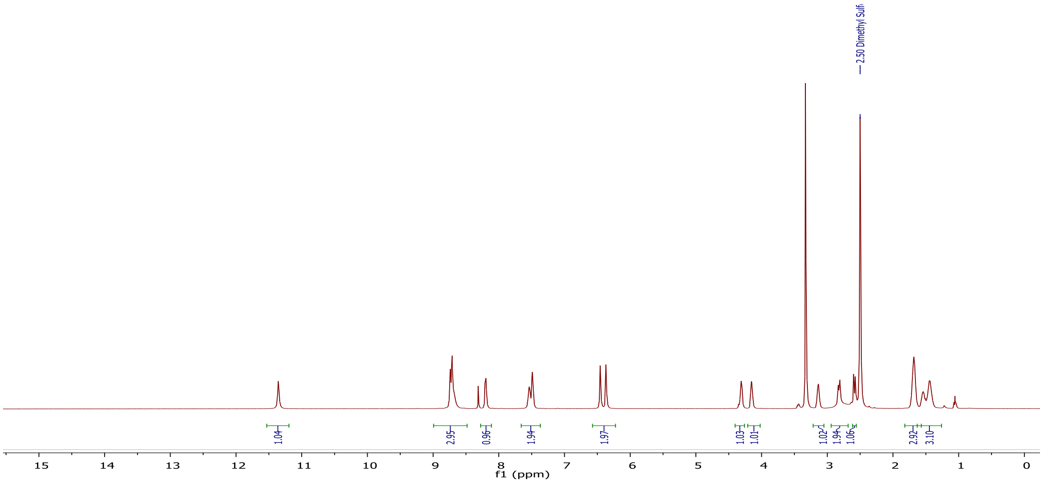

Figure S1. ^{1}H-NMR of **A** in DMSO-d_6.

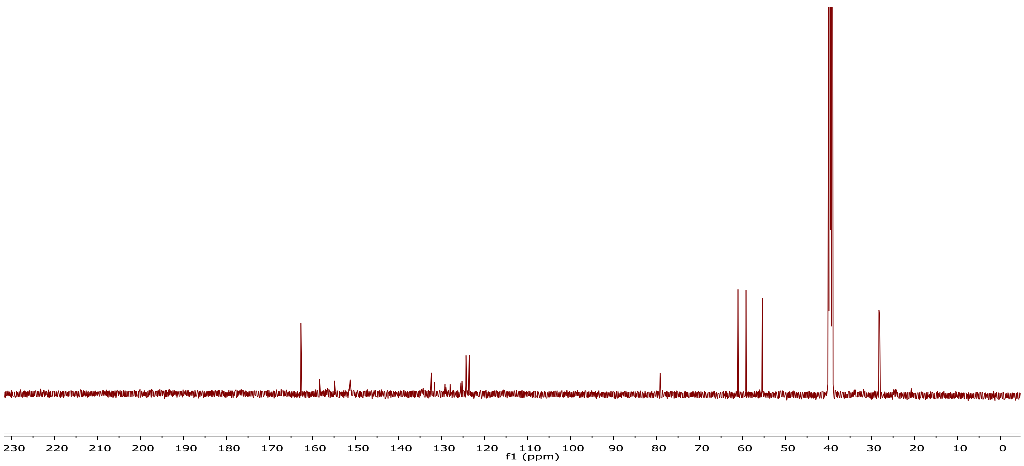

Figure S2. ^{13}C-NMR of **A** in DMSO-d_6.

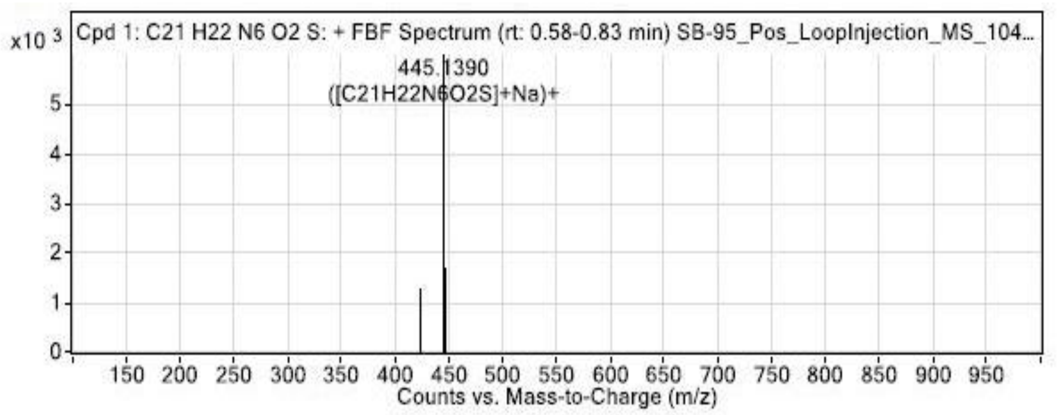

Figure S3. HR-MS spectrum of **A.**

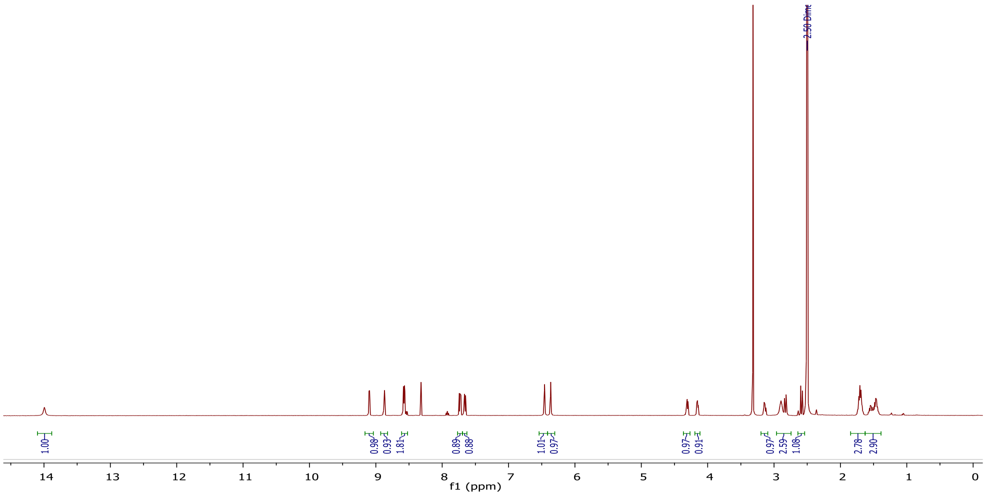

Figure S4. ^{1}H-NMR of **B** in DMSO-d_6.

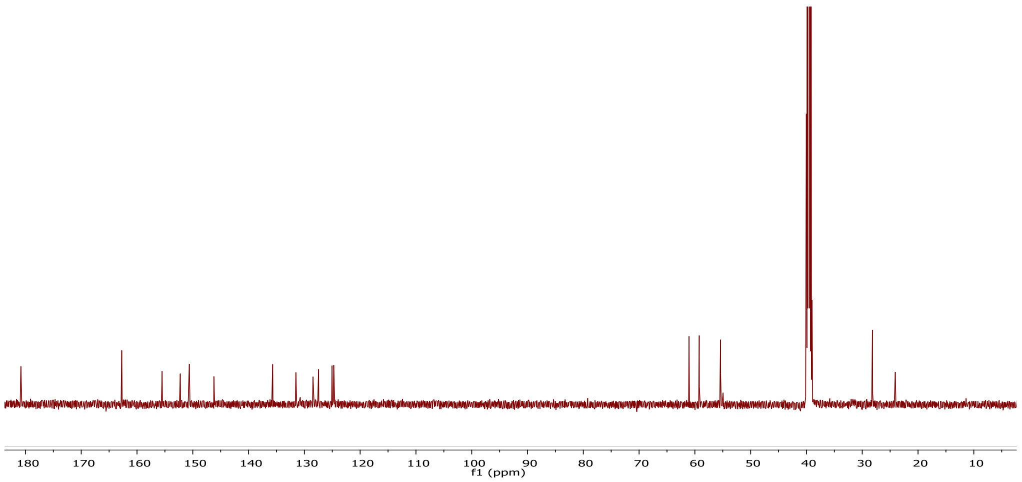

Figure S5. ^{13}C-NMR of B in DMSO-d_6.

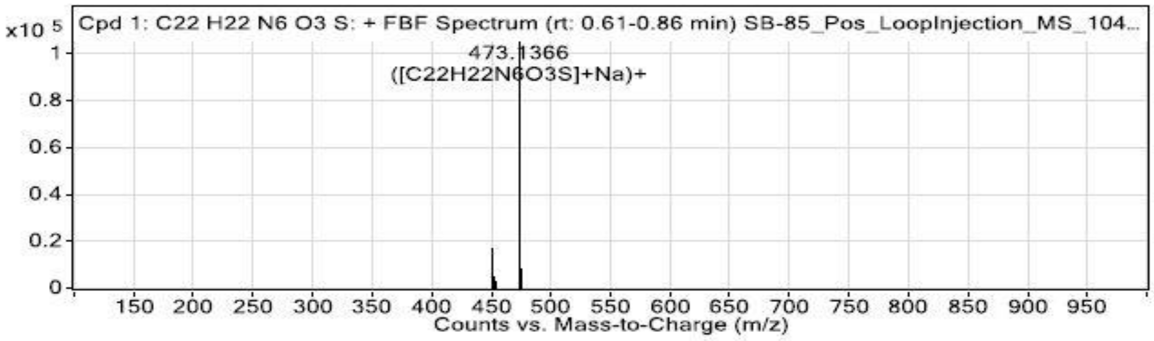

Figure S6. HR-MS spectrum of **B.**

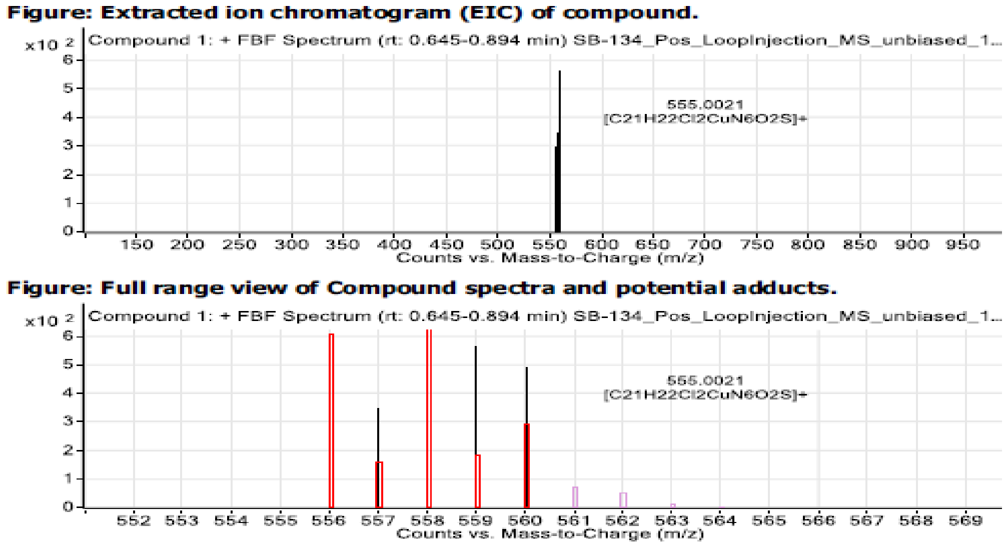

Figure S7. HR-MS spectrum of complex **1.**

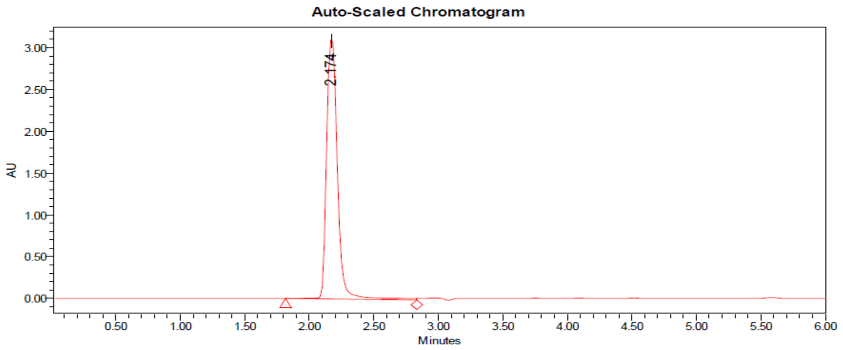

Figure S8. HPLC chromatogram of complex **1** in DMF/Acetontrile solution (HEPES buffer pH = 6.8).

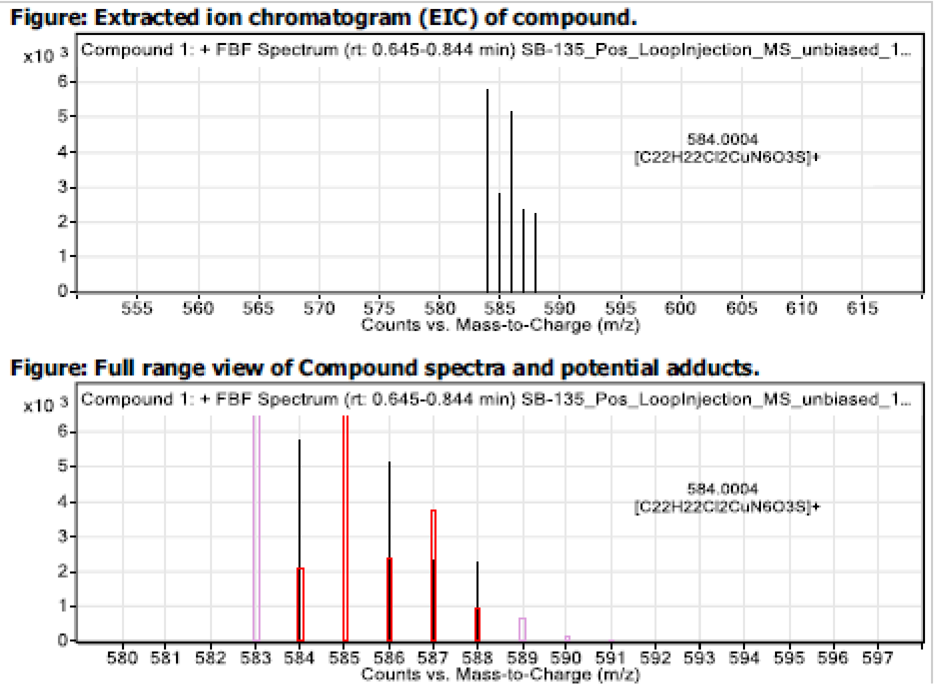

Figure S9. HR-MS spectrum of complex **2**.

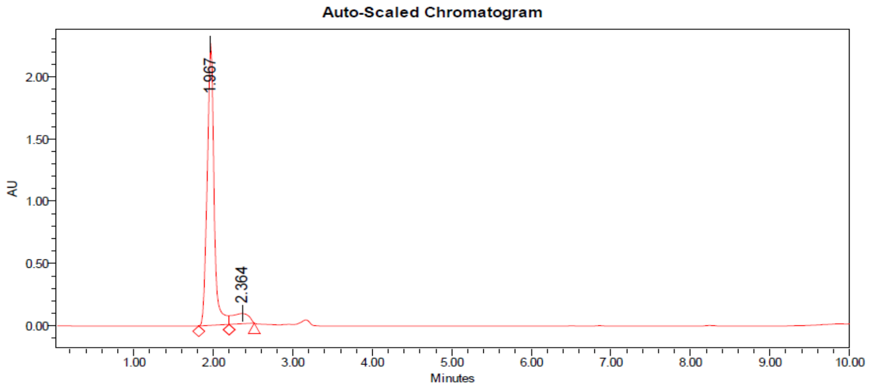

Figure S10. HPLC chromatogram of complex **2** in DMF/Acetontrile solution (HEPES buffer pH = 6.8).

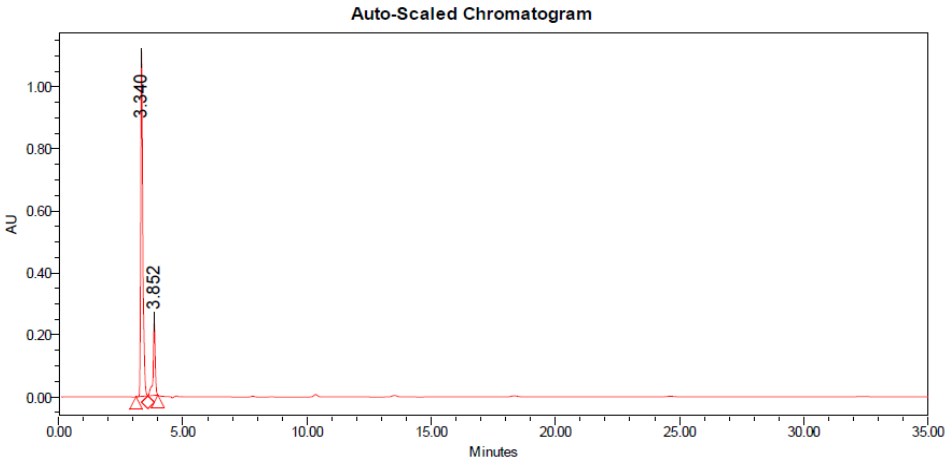

Figure S11. HPLC chromatogram of complex **3** in DMF/Acetontrile solution (HEPES buffer pH = 6.8).

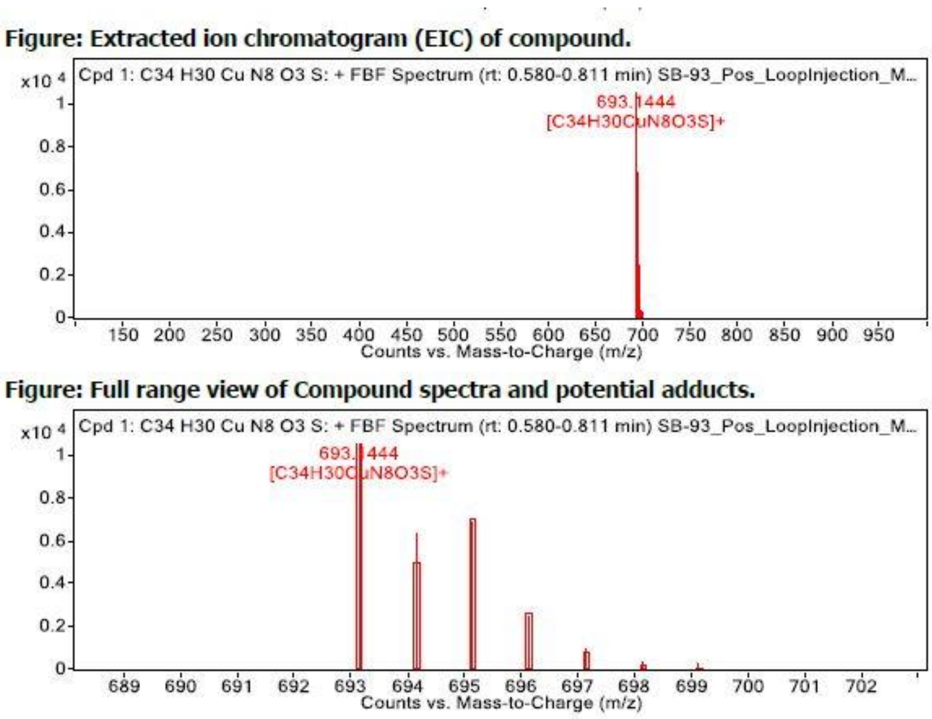

Figure S12. HR-MS spectrum of complex **4**

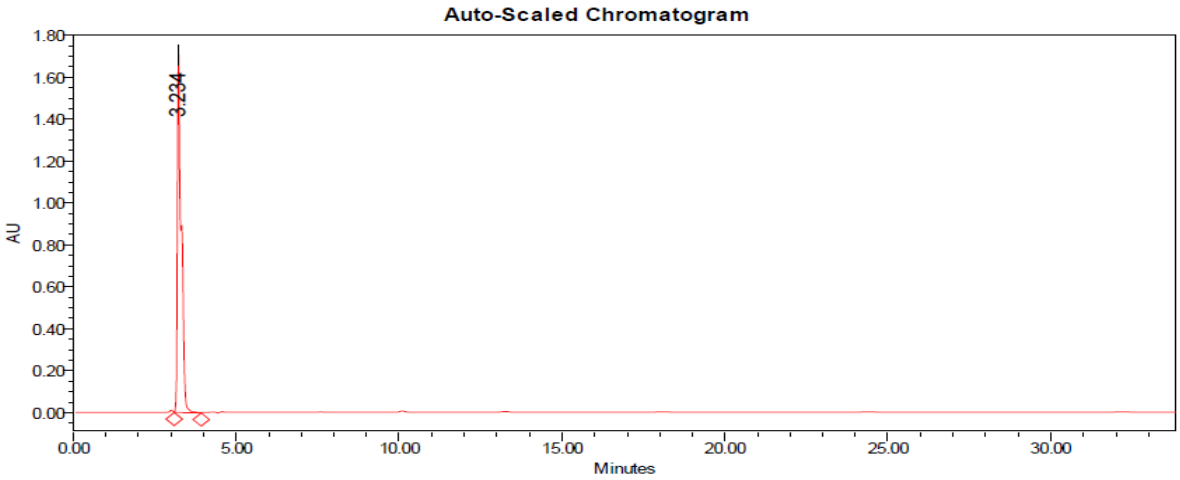

Figure S13. HPLC chromatogram of complex **4** in DMF/Acetontrile solution (HEPES buffer pH = 6.8).

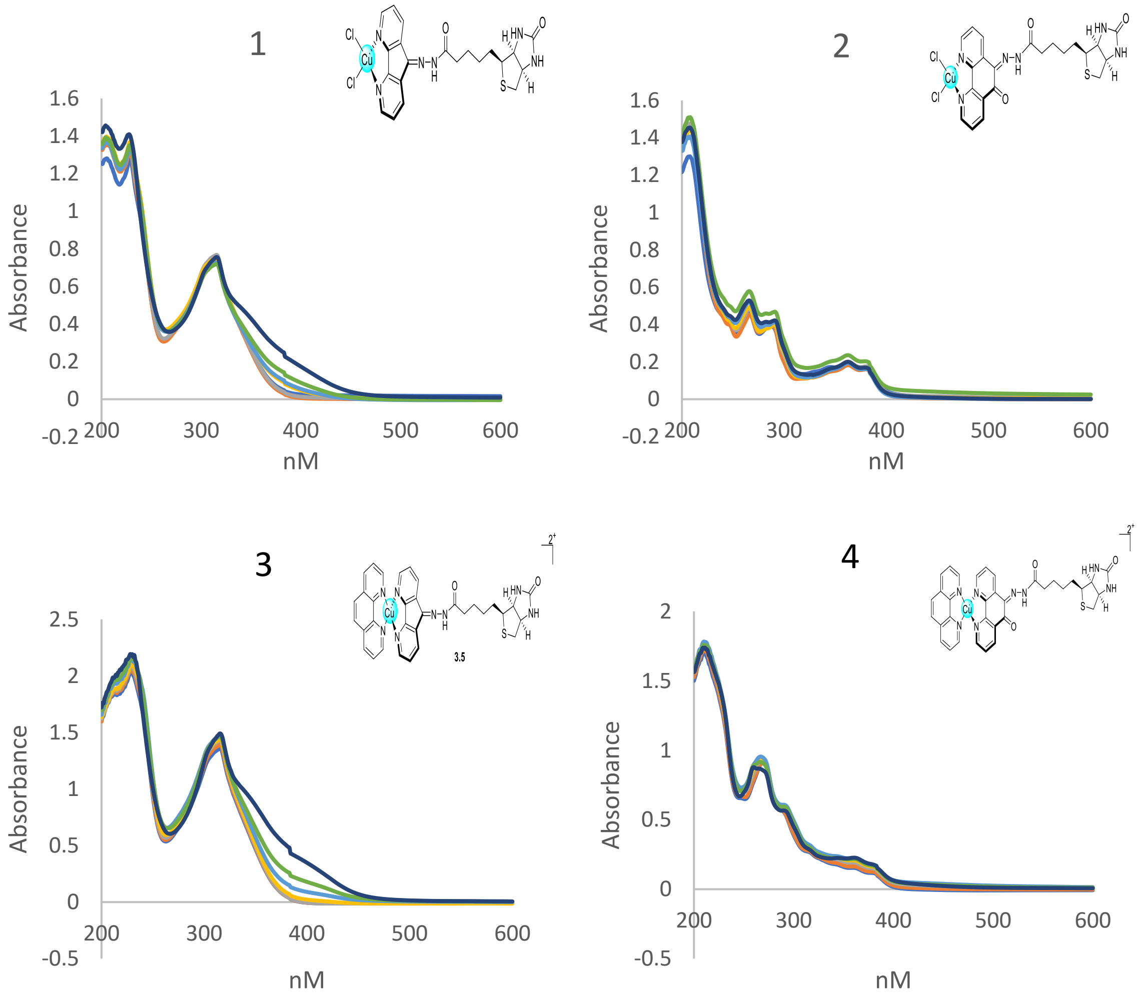

Figure S14. pH Dependence and Stability. UV VIS spectra of complexes **1-4** in the pH range 4-9. • pH = 4; • pH = 5; • pH = 6; • pH = 6 after 1 week; • pH = 7; • pH = 8; • pH = 9.

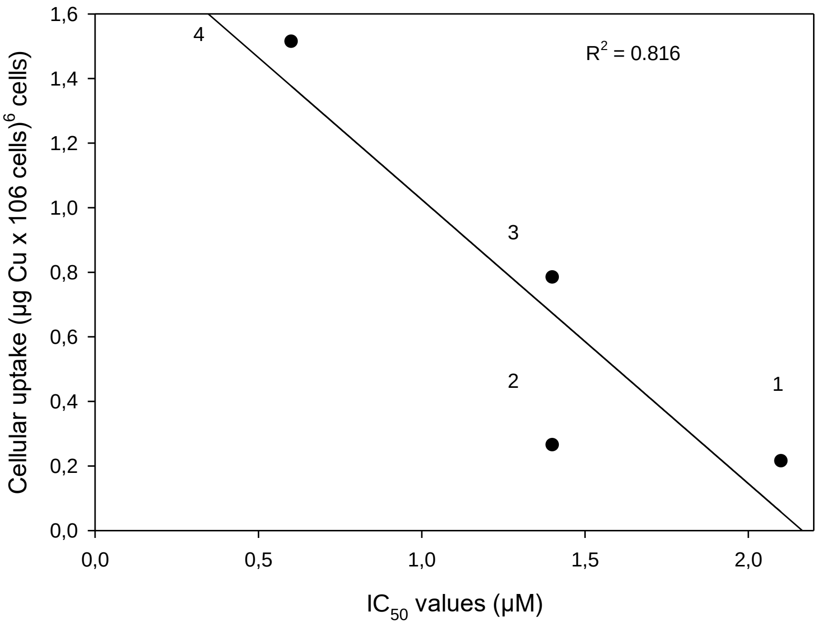

Figure S15. Correlation between cytotoxicity (IC_{50}) and cellular copper content in drug-treated MDA-MB-231 human cancer cells of complexes **1-4**.

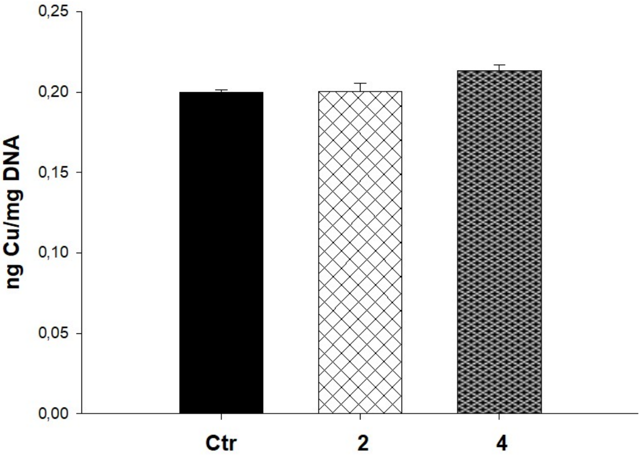

Figure S16. DNA metalation studies in MDA-MB-231 human cancer cells of complexes **2** and **4**. Cells (5 × 10^6) were seeded in 10 cm (diameter) Petri dishes in 10 mL of culture medium. Subsequently, cells were treated with 4 μM of tested complexes for 24 h. DNA was extracted and purified using a commercial spin column quantification kit (Qiagen DNeasy Blood and Tissue Kit). Only highly purified samples (A260/A230 ~ 1.8 and A280/A260 ~ 2.0) were included. Samples were then completely dried and re-dissolved in 200 μL of Milli-Q water (18.2 MW) for at least 20 min at 65 °C in a shaking thermo-mixer. Finally, samples were mineralized and the amount of cellular Cu was estimated by GF-AAS. Error bars are S.D.
